# Urinary metabolome at birth in patients with hypoxic–ischemic encephalopathy treated with therapeutic hypothermia and long-term neurodevelopmental outcomes: a 7-year follow up

**DOI:** 10.1186/s12967-025-06714-w

**Published:** 2025-11-24

**Authors:** Claudio Ancona, Enrico Valerio, Nicoletta Mainini, Alessio Favali, Ignazio D’Errico, Chiara Lasagni, Matteo Stocchero, Paola Pirillo, Giuseppe Giordano, Stefano Sartori, Eugenio Baraldi

**Affiliations:** 1 Pediatric Neurology and Neurophysiology Unit, Department of Women’s and Children’s Health, University of Padua, Padua, Italy; 2 Neonatal Intensive Care Unit, Department of Women’s and Children’s Health, University of Padua, Via Giustiniani 3, 35128 Padua, Italy; 3 Institute of Pediatric Research “Città della Speranza”, Padua, Italy; 4 Department of Neuroradiology, Azienda Ospedale-Università di Padova, Padua, Italy; 5 Department of Women’s and Children’s Health, University of Padua, Padua, Italy

**Keywords:** Hypoxic–ischemic encephalopathy, Newborn, Metabolomics, Long-term neurodevelopmental outcomes, Translational research

## Abstract

**Background:**

Hypoxic-ischemic encephalopathy (HIE) is a leading cause of neonatal mortality and morbidity, yet no validated biomarkers currently exist to predict long-term outcomes. We investigated the potential of the neonatal urinary metabolomic profile as a predictor of long-term neurodevelopmental outcomes in HIE newborns treated with therapeutic hypothermia (TH).

**Methods:**

We conducted a longitudinal study in neonates with HIE undergoing TH. Urine samples collected during TH were analyzed using untargeted metabolomics via mass spectrometry. Based on long-term follow-up outcomes, patients were categorized into two groups: the adverse outcome (AO) group, defined by perinatal death, cerebral palsy, and/or an intelligence quotient (IQ) < 70, and the favourable outcome (FO) group, defined as absence of CP and IQ ≥ 70. Additionally, we assessed the predictive value of early neonatal brain magnetic resonance imaging (MRI) in relation to the aforementioned outcomes.

**Results:**

Among 53 newborns treated with TH for HIE, long-term follow-up outcomes were available for 40; 29 were classified as FO and 11 as AO group. To mitigate bias, 11 FO patients were matched with 11 AO patients based on similar perinatal characteristics. Metabolomic analysis identified 21 metabolites distinguishing the two groups, with γ-butyrolactone, *N*-acetyl-galactosamine/glucosamine, Aldosterone, and Creatinine showing independent discriminative capability among groups. Brain MRI demonstrated a 67% positive and 96% negative predictive value for adverse outcomes.

**Conclusions:**

The identified metabolites are implicated in neuromodulation and neuronal susceptibility to damage, suggesting their potential as prognostic markers for long-term outcomes in HIE and warranting further investigation. This is the first study linking the acute-phase metabolomic profile with long-term neurodevelopmental outcomes in HIE neonates, supporting its prognostic potential.

**Supplementary Information:**

The online version contains supplementary material available at 10.1186/s12967-025-06714-w.

## Background

Perinatal asphyxia (PA) is the third leading cause of neonatal death worldwide, accounting for 23% of these fatalities [1]. Diagnosis is based on clinical criteria such as metabolic acidosis, an APGAR score below 5 at 10 min, and the need for invasive ventilation. The most severe consequence of PA is hypoxic-ischemic encephalopathy (HIE), whose clinical severity correlates with treatment efficacy and prognosis [2]. Currently, the only approved treatment for moderate to severe HIE is therapeutic hypothermia (TH), which must be initiated within six hours of life [3, 4]. TH significantly reduces mortality and major neurodevelopmental disabilities, with benefits evident not only at 18–24 months but also at 6–7 years of age [3–7]. Despite these advancements, the mortality rate for HIE remains around 30% during the neonatal period [7], and survivors are at high risk for long-term neurological sequelae, including cerebral palsy (CP), cognitive impairment, epilepsy, behavioral disorders, sensory dysfunction, and neurodevelopmental disorders [8]. Effective early prognostic tools are crucial for clinicians to identify children who need TH and to predict both short-term and long-term outcomes. However, no single tool is sufficient on its own. For instance, the combination of electroencephalography (EEG) background activity with post-rewarming brain magnetic resonance imaging (MRI) has shown the strongest predictive power for poor neurodevelopmental outcome, outperforming either prognostic tool used independently [9].

Conversely, to date, no biological prognostic markers for HIE have been validated. Metabolomics has emerged as an innovative approach to identifying early prognostic markers and therapeutic targets for various pathological conditions in newborns and children [10]. Few studies have investigated the urinary metabolome of healthy neonates compared to those with HIE [11, 12]. Previous research at our center used untargeted metabolomics analysis in newborns with HIE treated with TH, identifying two potential markers (l-lysine and l−3-methylhistidine) that may help in predicting the risk of HIE following PA [13]. Furthermore, significant alterations were observed in pathways related to neurosteroidogenesis, lysine degradation, and carnitine synthesis in HIE patients during their first 96 h of life [14]. However, the relationship between the urinary metabolomic profile of HIE patients treated with TH and long-term adverse outcomes has not yet been investigated.

Therefore, this study aimed to determine whether a specific metabolomic fingerprint at birth correlated with favourable or adverse long-term neurodevelopmental outcome in a cohort of patients receiving TH for HIE. We compared patients with major adverse outcomes (perinatal death, CP development, and/or intelligence quotient, IQ, < 70) to those with favourable outcome, defined as absence of CP and IQ ≥ 70. To our knowledge, this is the first study to explore the correlation between the neonatal urinary metabolome and long-term outcomes in newborns with HIE.

Additionally, we evaluated the association between brain injury observed in early post-rewarming MRI and major adverse outcomes.

## Methods

### Patients and study design

We conducted a longitudinal single-center study, enrolling infants born at > 35 weeks of gestation with HIE secondary to PA who underwent TH according to international and national guidelines [4, 14, 15]. Inclusion criteria were: gestational age of 35 weeks or greater; evidence of peripartum asphyxia, with the presence of at least one of the following criteria: Apgar score of 5 or less at 10 min, mechanical ventilation or resuscitation at 10 min, cord pH < 7.0, or an arterial pH < 7.0 or base deficit ≥ 12 within 60 min of birth; evidence of moderate or severe encephalopathy according to modified Sarnat score; [5] and absence of major congenital abnormalities. Patients were admitted to the neonatal intensive care unit (NICU) of the Department of Women's and Children's Health at the University Hospital in Padova, Italy, from May 2015 to April 2021. For each patient, we recorded gestational age, birth weight, sex, Apgar scores at 1, 5, and 10 min, delivery mode, need for neonatal resuscitation, type and severity of organ failure, Sarnat Score at 60 min of life, pH, base excess (BE) from cord blood and/or a blood sample at 1 h of life, and pharmacological treatment. All patients underwent neurophysiological monitoring with amplitude-integrated EEG (aEEG), and at least 1-h full video EEG (vEEG) was performed.

The study was approved by the local Ethics Committee (Azienda Ospedale-Università di Padova, reference 4332/AO/17).

### Sampling

For each patient, written informed parental consent was obtained by means of a specific consent form. Afterwards, urine samples were collected before and during TH and in the post-rewarming phase, with the previously described methodology [13]. Samples were stored at − 80 °C until analysis.

### Metabolomic analysis

Protocol for untargeted metabolomics analysis has been described in detail previously [13]. Metabolomic analysis was performed at the Mass Spectrometry and Metabolomics Laboratory of the Institute of Pediatric Research, at the Women's and Child’s Health Department at Padova University. Protocol for untargeted metabolomics analysis, data pre-processing and variable annotation have been described in detail previously [13].

### Brain MRI investigation

All infants included in the study underwent early brain MRI after rewarming at a median age of 6 days. The MRI equipment was a Philips Achieva 1.5 Tesla (Philips Healthcare, Best, Netherlands). The MRI sequence protocol included 3D gradient echo T1-weighted images, gradient echo T2-weighted images, turbo spin echo T2-weighted images, and echo planar diffusion-weighted images. MRI scans were blindly reviewed by an experienced paediatric neuroradiologist, and damage was graded according to the score proposed by Barkovich et al. [16]. Each patient was assigned a score based on the extent of damage to the basal ganglia, cortical watershed areas, and the combined damage to both. Patients with a cumulative score > 0 were considered to have a brain injury on neuroimaging.

### Follow-up clinical assessment and WPPSI-IV evaluation

At our centre, patients with HIE undergo regular evaluations as part of a follow-up program until school age (6–7 years). According to study protocol, control visits were conducted between April and September 2023, including cognitive development assessments using the Wechsler Preschool and Primary Scale of Intelligence—4th edition (WPPSI-IV). The WPPSI-IV measures cognitive abilities in children aged 2.6–7.7 years through 15 subtests, generating composite scores for specific cognitive domains (e.g., verbal comprehension, working memory) and a total IQ score [17, 18]. WPPSI-IV was administered in patients meeting the inclusion criteria for test administration (in particular age between 2 years and 6 months and 7 years and 7 months). During follow-up visits, patient's health and development were assessed, including details on past or ongoing rehabilitation programs. A complete neurological examination was conducted to detect signs of CP and minor neurological dysfunction. The WPPSI-IV was administered by an experienced psychologist blinded to the patients'clinical histories. Patients were categorized into two groups: the adverse outcome (AO) group (defined as the major adverse outcomes of perinatal death, or CP, and/or IQ < 70) and the favourable outcome (FO) group (defined as absence of CP and IQ ≥ 70). No cases of patients with CP and IQ ≥ 70 were observed at follow-up. Patients previously diagnosed with permanent global neurological impairment were excluded from the WPPSI-IV assessment due to feasibility issues and were assigned by default in the AO group; the same was done for newborns who died in the neonatal period from secondary HIE damage and/or were redirected to comfort care within the very first days of life due to the extreme severity of their clinical condition and/or electroencephalographic pattern. Neonatal brain MRI results were compared between the AO and FO groups to determine the early MRI's predictive value for major adverse outcomes.

### Statistical data analysis

Characteristics of the recruited infants were analysed using the t-test or Mann–Whitney test for continuous normally or non-normally distributed data, respectively, and by Fisher’s exact test for categorical variables. A significance level a = 0.05 was assumed. Normality of the data was assessed by Shapiro–Wilk test (p > 0.10). A one-to-one matching procedure was applied to extract groups of newborns without significant differences in the demographic and clinical perinatal data, to avoid bias in the metabolomics data analysis due to confounding effects. A significant level of α = 0.05 was assumed in the procedure. Metabolomics data were investigated by multivariate data analysis based on projection methods. Specifically, Principal Component Analysis (PCA) was used for outlier detection and orthogonally constrained Partial Least Squares (PLS) for classification (oCPLS2C) with stability selection [19–21], for discovering metabolomics features promising in distinguishing patient groups independently of the effects of gestational age and birth weight. Multiple Linear Regression (MLR) was applied to better characterize the effect of the patient group on the selected metabolomics features. Data analysis was performed using in-house R-functions implemented by R 4.0.4 platform (R Foundation for Statistical Computing). More details about statistical data analysis can be found in the Supplementary Information.

## Results

From May 2015 to April 2021, 53 neonates with HIE treated with TH were enrolled in our study cohort. Follow-up outcomes, including perinatal death, CP, and/or an IQ below 70, were available for 40 patients.

**Fig. 1:**
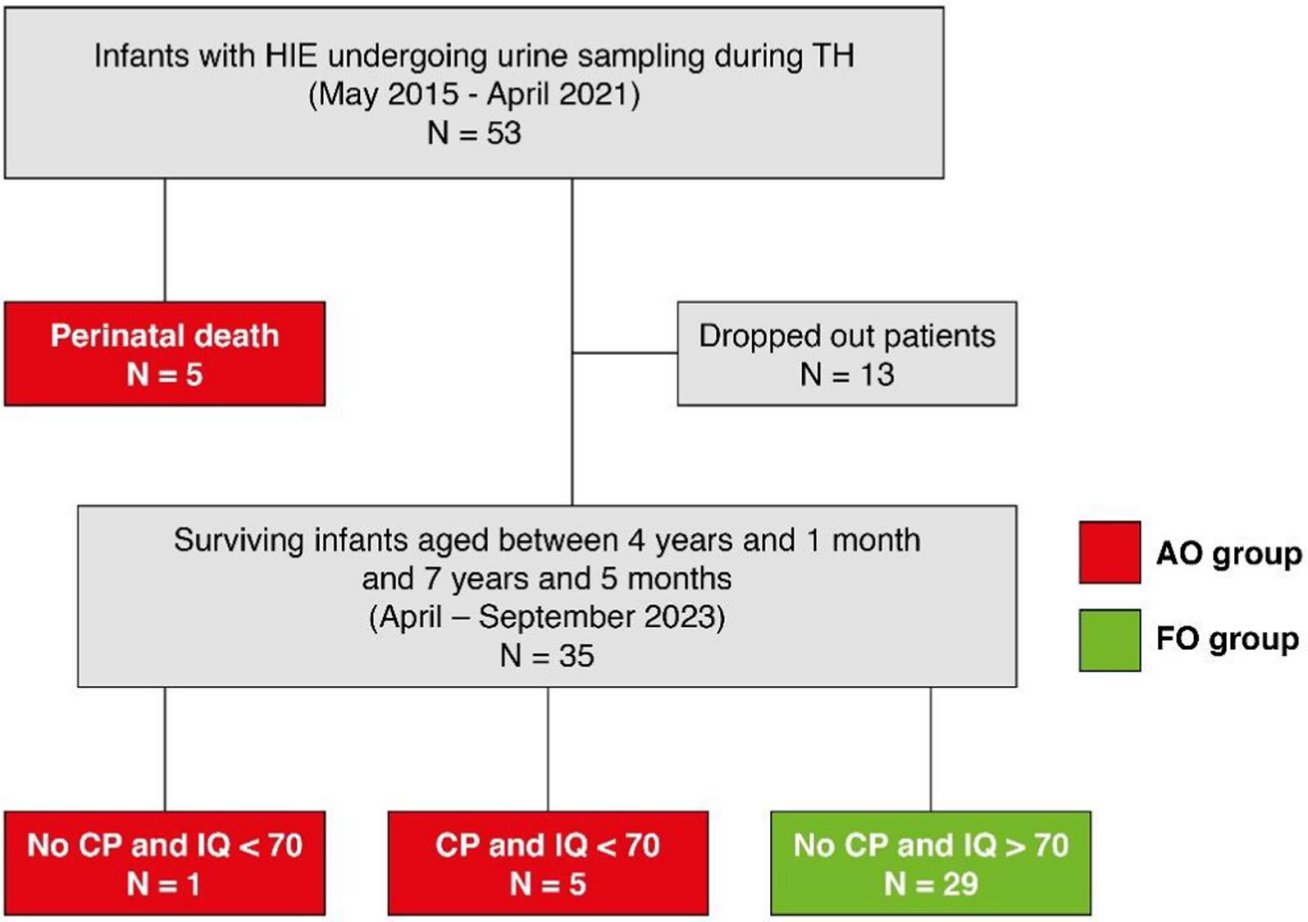
Outcome of patients recruited in the study. Dropouts: patients for whom follow-up data were not available. AO group: adverse outcome group FO group: favourable outcome group

Among these, 29 were classified in the FO group, while 11 were classified in the AO group. Within the AO group, 5 infants died during the perinatal period, 5 developed CP and had an IQ below 70, and 1 had an IQ below 70 without CP. There were no patients with CP and an IQ above 70 in this cohort. 13 patients were lost to follow-up, and it was not possible to assess them clinically or obtain clinical information via telephone contact (Fig. 1).

From a neuroradiological perspective, 26 infants were classified as having no damage at brain MRI, with 25 (96.2%) of these belonging to the favourable outcome group. Interestingly, among those with radiologically identifiable brain damage (cumulative score > 0 according to Barkovich et al.), 4 belonged to the FO group (28.6%), and 10 to the AO group; 5 of the latter developed CP and had an IQ < 70 (35.7%), while 5 died during the perinatal period (35.7%). In our cohort, the positive predictive value of early brain MRI for major adverse outcomes was 67%, while the negative predictive value was 96%.

Since the urinary metabolome is strongly dependent both on the gestational age and on the birth weight, a matching procedure was applied to avoid bias in data analysis. As a result, 11 newborns were selected in the FO group to match the 11 newborns of the AO group on the basis of the demographic and clinical perinatal data. The study follow-up visits were conducted between April and September 2023. Patients’ age at the last follow-up ranged from 4 years and 1 month to 7 years and 5 months. Specifically, a follow-up duration exceeding 5 years was observed in 83% of AO patients and 67% of FO patients. 17% of patients in the AO group and 4% in the FO group reached a 7-year follow-up.

In Table 1, the clinical characteristics of the matched subjects are summarised.

**Table 1:** Characteristics of the matched newborns

Characteristics	Favourable outcome (FO) Group n = 11	Adverse outcome (AO) Group n = 11	p
Sex M (F)	4 (7)	8 (3)	0.20
Gestational age in days, m (SD)	276.4 (13.6)	270.9 (12.2)	0.25
Birth weight m (SD)	3345 (463)	3013 (747)	0.18
Delivery mode vaginal (caesarean)	6 (5)	4 (7)	0.67
SARNAT 60 min M [IQR]	2 [0]	2 [1]	0.18
Apgar 1 min M [IQR]	3.0 [2.5]	2 [2]	0.57
Apgar 5 min M [IQR]	5 [3]	4 [3]	0.62
Apgar 10 min M [IQR]	7.0 [3.5]	5 [2]	0.51
Hypoglycemia on admission n (y)	9 (2)	6 (5)	0.36
pH m (SD)	7.00 (0.19)	7.09 (0.22)	0.32
BE M [IQR]	− 13.7 [8.3]	− 16.6 [9.3]	0.79
pH a 1 h m (SD)	7.16 (0.18)	7.12 (0.15)	0.41
EB a 1 h M [IQR]	− 16.0 [8.5]	− 18.3 [8.6]	0.32
EOS n (y)	4 (7)	5 (6)	1.00
LOS n (y)	8 (3)	6 (5)	0.66
Resuscitation [1] n (y)	1 (10)	1 (10)	1.00
Resuscitation [2] n (y)	8 (3)	7 (4)	1.00
Resuscitation [3] n (y)	7 (4)	7 (4)	1.00
ATB n (y)	0 (11)	0 (11)	1.00
Inotropes n (y)	6 (5)	7 (4)	1.00
Phenobarbital n (y)	5 (6)	0 (11)	0.04
Phenytoin n (y)	11 (0)	8 (3)	0.21
Benzodiazepines n (y)	11 (0)	9 (2)	0.48
Red blood cells suppl. n (y)	9 (2)	6 (5)	0.36
Plasma suppl. n (y)	1 (10)	2 (9)	1.00
Platelets suppl. n (y)	8 (3)	7 (4)	1.00
MOF [1] n (y)	5 (6)	7 (4)	0.67
MOF [11] n (y)	11 (0)	9 (2)	0.48
MOF [2] n (y)	3 (8)	2 (9)	1.00
MOF [3] n (y)	6 (5)	5 (6)	1.00
MOF [33] n (y)	6 (5)	7 (4)	1.00
MOF [4] n (y)	3 (8)	4 (7)	1.00

Normally distributed data are reported as means (SD), non-normally distributed data as medians [25th–75th], and categorical variables as numbers of occurrences (n: no, y: yes); p is the p-value of the statistical test applied. GA: Gestational Age; BE: Base Excess; EOS: Early Onset Sepsis; LOS: Late Onset Sepsis; ATB: antibiotic therapy; MOF: multiorgan failure; MOF [1]: increased creatinine; MOF [11]: increased creatinine plus oliguria; MOF [2]: elevated transaminases; MOF [3]: increased troponin I; MOF [33]: use of inotropes with or without elevated troponin I; MOF [4]: coagulopathy

**Fig. 2:**
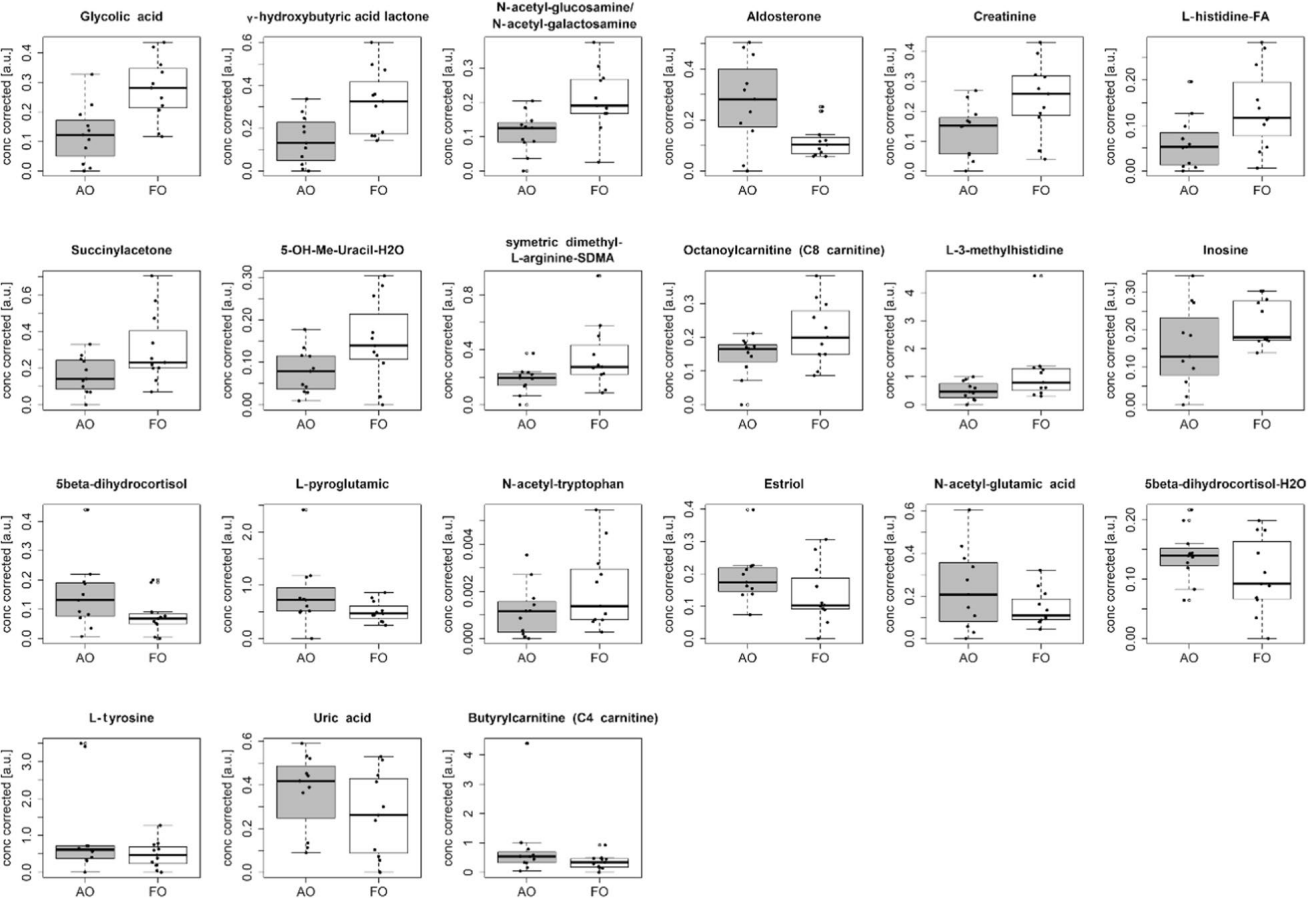
Box plots of metabolites relative concentration. It shows the distributions of the relative concentration corrected by gestational age and birth weight of the selected metabolites between AO (grey) and FO (white) groups

Metabolomic analysis led to a data set composed of 96 metabolomic features, 25 generated using the negative ionization mode and 71 using the positive ionization mode. Only the first urine sample available for each patient was considered in data analysis. No outliers were detected by PCA considering the T2 and Q-test assuming a level of significance of 0.05. As a consequence, a data set of 22 observations and 96 features was investigated (Fig. 2).

As a first step of data analysis, the optimal subset of metabolomic features to distinguish the FO group and the AO group was discovered by oCPLS2C with stability selection.

Twenty one out of the 96 features resulted to be the most relevant features in characterising the differences between the two groups, and were included in the optimal subset. Thus, the oCPLS2C classification model obtained considering the most relevant features was built. The model showed 1 score component, MCC = 0.647, MCCcv = 0.567, MCC for the out-of-bag prediction during stability selection equal to 0.500 and passed the permutation test on the group. To better investigate the effect of the group on the urinary metabolome, MLR analysis correcting the model for gestational age and birth weight, was applied. Five out of the 21 features (Glycolic acid, γ-butyrolactone, *N*-acetyl-galactosamine/glucosamine, Aldosterone, and Creatinine) showed a significant effect of the group (p < 0.05), as reported in Table 2.

**Table 2:** Selected relevant metabolites

m/z	Rt	Annotation	p
75.0077	0.6126	Glycolic acid	0.005
85.0286	0.6126	γ-hydroxybutyric acid lactone	0.011
222.0977	0.6939	*N*-acetyl-glucosamine/*N*-acetyl-galactosamine	0.017
361.2020	5.7591	Aldosterone	0.023
114.0672	0.5912	Creatinine	0.042
110.0723	0.6614	l-Histidine-FA	0.053
157.0501	3.1623	Succinylacetone	0.061
125.0353	0.7729	5-OH-Me-Uracil-H2O	0.065
203.1511	0.6074	Symetric dimethyl-l-arginine-SDMA	0.071
288.2175	5.2407	Octanoylcarnitine (C8 carnitine)	0.074
170.0935	0.5804	l−3-methylhistidine	0.132
267.0731	1.4044	Inosine	0.136
365.2327	5.8294	5beta-dihydrocortisol	0.146
130.0509	0.9817	l-Pyroglutamic	0.153
245.0925	4.7709	*N*-acetyl-tryptophan	0.193
289.1824	5.2570	Estriol	0.206
188.0558	1.0463	*N*-acetyl-glutamic acid	0.208
347.2224	5.9895	5beta-dihydrocortisol-H2O	0.217
180.0660	1.0680	l-tyrosine	0.227
167.0205	0.8430	Uric acid	0.256
232.1555	2.4030	Butyrylcarnitine (C4 carnitine)	0.259

m/z is the mass to charge ratio, Rt is the retention time in minutes, annotation is the name of the annotated metabolite and p is the p-value of the factor group calculated by MLR analysis

## Discussion

This study explored the correlation between the urinary metabolome at birth and both short- (i.e., death) and long-term major adverse neurodevelopmental outcomes (defined as the development of cerebral palsy and/or an IQ below 70) in a cohort of patients with HIE treated with TH. To our knowledge, this is the longest follow-up study to date evaluating this correlation.

A strength of this study is the implementation of a strict matching of demographic variables between the AO and FO patient cohorts. Given that metabolomic analysis is highly dependent on gestational age and weight variability, this rigorous matching allowed for the generation of robust results in terms of key metabolites. These findings were further supported both by the neuroprotective and neuromodulatory role played by some of the identified metabolites, and by the recurrence of some of them—specifically, l−3-methylhistidine—consistent with previous studies conducted by our group [13, 14].

Untargeted urinary metabolomics identified five independent relevant discriminant metabolites, with an additional 16 metabolites retaining discriminative capability only when considered collectively. The five metabolites with independent discriminant capability were searched in the Human Metabolome Database [22] and compared with literature data. Four of them were found to have biological plausibility: γ-butyrolactone, N-acetyl-galactosamine/glucosamine, Aldosterone, and Creatinine*.*

γ-butyrolactone, a precursor of γ-hydroxybutyrate (an endogenous neurotransmitter with neuromodulatory GABAergic properties) [23], can decrease the excitatory glutamatergic functions of NMDA and AMPA/Kainate receptors [24]. AO group showed lower levels of urinary γ-butyrolactone; this may indicate a reduced neuromodulatory tone, leading to an imbalance toward excitotoxicity.

*N*-acetyl-galactosamine and glucosamine, amino derivatives of galactose and glucose, are constituents of tissue glycoproteins, contributing to various cellular functions, including protein stability, transcription, mitochondrial function, and cell survival [22]. In our cohort, urinary levels of these metabolites were lower in AO group, and this may be due to increased consumption during severe HIE insult. This finding aligns with prior research indicating lower urinary levels of these aminoacids in newborns with HIE compared to healthy controls [25].

Aldosterone, a mineralocorticoid primarily regulating blood sodium and potassium balance, also impacts central nervous system cells [26]. Activation of mineralocorticoid receptors can downregulate calcium channel and NMDA glutamatergic receptor expression, reducing neuronal excitotoxicity, and increase expression of anti-apoptotic proteins Bcl-2 and Bcl-xl [27]. Urinary aldosterone levels were elevated in AO group, suggesting a positive feedback mechanism of a defence antiapoptotic response to acute hypoxic injury.

Creatinine, derived from creatine/phosphocreatine energy metabolism and eliminated via glomerular filtration, showed lower levels in the AO group, possibly due to more severe systemic hypoxic damage and reduced glomerular filtration rate (GFR). Despite similar incidences of renal failure between the AO and FO groups, the AO group may have experienced greater reductions in GFR, resulting in lower urinary creatinine levels.

Among the metabolites that collectively retain discriminative capability, pipecolic acid and l−3-methylhistidine deserve a mention.

Pipecolic acid is a catabolite of intact lysine. Intact lysine is important for neuroprotection, anti-inflammatory processes, and stress response modulation [28–30]. Previous research by our group [12] found elevated urinary levels of intact lysine in HIE patients without neuroimaging-detected brain injury, possibly indicating reduced degradation of this neuroprotective amino acid, witnessing a better adaptive response. Additionally, Piñeiro-Ramos et al. found increased metabolites of the lysine degradation pathway in brain-injured patients [31, 32]. In our study, higher urinary pipecolic acid concentrations in the adverse outcome (AO) group suggest increased lysine degradation and reduced neuroprotection.

l−3-methylhistidine is released during the degradation of muscular actin and myosin peptide chains. Both animal and human studies have identified l−3-methylhistidine as a marker of kidney injury, with urinary excretion inversely correlated with the severity of glomerular filtration impairment [33, 34]. Our previous research identified l−3-methylhistidine as an indicator of hypoxic-ischemic brain injury on MRI, with lower concentrations in affected patients [12]. In this study, its levels were lower in the AO group. In HIE, L-3-methylhistidine levels may reflect the extent of glomerular filtration impairment due to hypoxic injury, potentially predicting both short-term (MRI-detected brain injuries) and long-term outcomes in neonates with HIE.

Discussed metabolites are involved in various metabolic pathways associated with HIE brain injury. Their alteration correlate with the production of reactive oxygen species, decreased antioxidant capacity, mitochondrial energy deficit, neuronal excitotoxicity, reduced neuroprotection, enhanced apoptotic processes, and multiorgan damage [35, 36]. The discussed metabolites deserve further investigation as they potentially represent early markers for long-term neurological outcomes in children with neonatal HIE.

Interpreting the results not only from a prognostic but also a potential therapeutic perspective, the trends of γ-hydroxybutyrate and pipecolic acid in the AO and FO groups, considered in light of the well-documented neuromodulatory and neuroprotective properties of γ-hydroxybutyrate and intact lysine, respectively, may suggest the potential utility of supplementing the latter as adjuvant treatments to therapeutic hypothermia in newborns with HIE.

We also evaluated the predictive value of brain injury detected by early post-rewarming MRI scans for major adverse outcomes (perinatal death, or CP, and/or IQ < 70). In our cohort, early MRI performed shortly after rewarming (median age of 6 days) showed a positive predictive value of 67% and a negative predictive value of 96%. Early MRI damage score, based on the Barkovich classification, seemed to predict a favourable prognosis in infants without brain injuries, while its correlation with major adverse outcomes was weaker. However, the small cohort size limits the statistical significance of this finding.

Commonly, brain MRI is performed soon after rewarming to visualize DWI sequence alterations, characterize injury extent and timing, and formulate a prognosis. However, early MRI scans may inaccurately estimate injury severity [37–39]. Consequently, the American College of Obstetrics and Gynecology and the American Academy of Pediatrics recommend two brain MRIs in the follow-up of HIE infants: one in the first week to determine injury timing and another after the first week to fully evaluate injury extent [40]. However, conducting two brain MRIs in a short timeframe presents challenges, including the need for repeated sedation.

Integrating early MRI findings with other diagnostic and prognostic tools—such as MRS (magnetic resonance spectroscopy) and EEG—can enhance early outcome predictions [8]. In this context, a clinical laboratory science approach, incorporating also metabolomic data, may further improve prognostic accuracy [41]. We believe that, given the substantial amount of data acquired on the early metabolomic profile in large cohorts of asphyxiated patients with encephalopathy, machine learning techniques will enable the development of rapid bedside tests in the near future, capable of analyzing the global metabolic profile ("fingerprint") of a given newborn with HIE to predict their outcome. The advent of such prognostic tool could further improve early prognostic characterization, enabling tailored acute care and aiding clinicians in the decision to initiate TH, ultimately allowing an individualized follow-up.

In fact, our findings propose a conceptual model integrating amplitude-integrated or full EEG ((a)EEG) background activity analysis, early brain MRI, and urinary metabolomic profile. If derived data are coherent (i.e., showing a severely abnormal (a)EEG background activity, significant brain injury at early brain MRI, and suggestive metabolome alterations at birth), proposed model can be considered as a reliable combined prognostic tool for subsequent abnormal neurodevelopment without needing repeated MRI. Conversely, if data are discordant (e.g., normal (a)EEG background activity and metabolomic profile but early MRI showing brain injury), a follow-up MRI post-acute phase might better characterize brain injury without overestimation (see Supplementary Fig. 1).

The number of patients in AO group was smaller than in the FO group, which could have reduced the statistical power. Similarly to other studies, we considered only major adverse outcomes [4, 5] focusing on perinatal death or the development of CP and/or IQ < 70. Expanding the study size would increase the number of patients in the AO group and allow for a more detailed assessment of long-term outcomes, including differentiation between mild, moderate, and severe outcomes.

It is also to be noted that, since this is an exploratory study and there was not preliminary pilot data to use for power analysis, it was not possible to a-priori estimate the sample size useful to obtain a given level of statistical power. On the other hand, it is acknowledged that retrospective power analysis is not a reliable approach, as the calculated observed power correlates with the p-value and does not provide any additional meaningful information.

Although urine sampling is non-invasive, comparative plasma studies are necessary to assess metabolite concentrations across different body fluids and to gain a more comprehensive understanding of their metabolism in infants with HIE. Additionally, diagnosing intellectual disability requires more than WPPSI-IV performance, necessitating adaptive functioning assessments. Future studies should incorporate psychometric evaluations with adaptive functioning tools.

## Conclusions

To our knowledge, this is the longest follow-up study correlating the urinary metabolome at birth with subsequent neurodevelopment in patients receiving TH after HIE, allowing us to identify a metabolomic profile associated with adverse long-term outcomes. The potential neuroprotective role of some of the metabolites identified has already been validated, both from our previous results and from studies conducted on animal models of HIE [42–44]. In this frame, our study acts as a proof of concept – demonstrating conceptual validity and biological consistency of our results, which must be reproduced in the future and confirmed through expansion of the original patient cohort and further targeted metabolomic investigations. This, in turn, could provide further clues regarding the usefulness of supplementing neuromodulatory metabolites as an adjunct treatment to TH in neonates with HIE, as already existing literature strongly suggests. Furthermore, validation of these exploratory findings through future studies employing targeted metabolomics may confirm the role of potential biomarkers that, combined with early brain MRI, can enhance the predictive accuracy of long-term outcomes.

Ultimately, the effort made by our team and all other research groups around the world in translational research related to hypoxic-ischemic encephalopathy may lead to further improvement in the long-term outcomes of these patients, generating a global health benefit for future generations [45].

## Supplementary Information

Supplementary Material 1: Figure 1: chart for the indication of late MRI in HIE. This hypothetical conceptual chart aims to assist clinicians in assessing the need for a follow-up late brain MRIby integrating findings fromEEG background analysis, neonatal metabolomic profile, and early brain MRI

Supplementary Material 2

## Data Availability

The data sets generated and/or analysed during the current study are publicly available at the following link: https://data.mendeley.com/datasets/xx429fc953/1.

## Abbreviations

aEEG: Amplitude-integrated EEG
AMPA: α-Amino-3-hydroxy-5-methyl-4-isoxazolepropionic acid
AO group: Adverse outcome group
CP: Cerebral palsy
EEG: Electroencephalography
FO group: Favourable outcome group
GABA: Gamma-aminobutyric acid
GFR: Glomerular filtration rate
HIE: Hypoxic–ischemic encephalopathy
IQ: Intelligence quotient
MCC: Matthews correlation coefficient
MLR: Multiple linear regression
MRI: Magnetic resonance imaging
NICU: Neonatal intensive care unit
NMDA: *N*-Methyl d-aspartate
PA: Perinatal asphyxia
PCA: Principal component analysis
PLS: Partial least squares
TH: Therapeutic hypothermia
vEEG: Video EEG
WPPSI-IV: Wechsler preschool and primary scale of intelligence 4Th edition
