# Supplementary figures and images for: Urinary metabolome at birth in patients with hypoxic–ischemic encephalopathy treated with therapeutic hypothermia and long-term neurodevelopmental outcomes: a 7-year follow up
Source: J Transl Med. 2025 Nov 24;23:1345. doi: 10.1186/s12967-025-06714-w (PMC12645678; doi:10.1186/s12967-025-06714-w)

# LATE MRI INDICATION

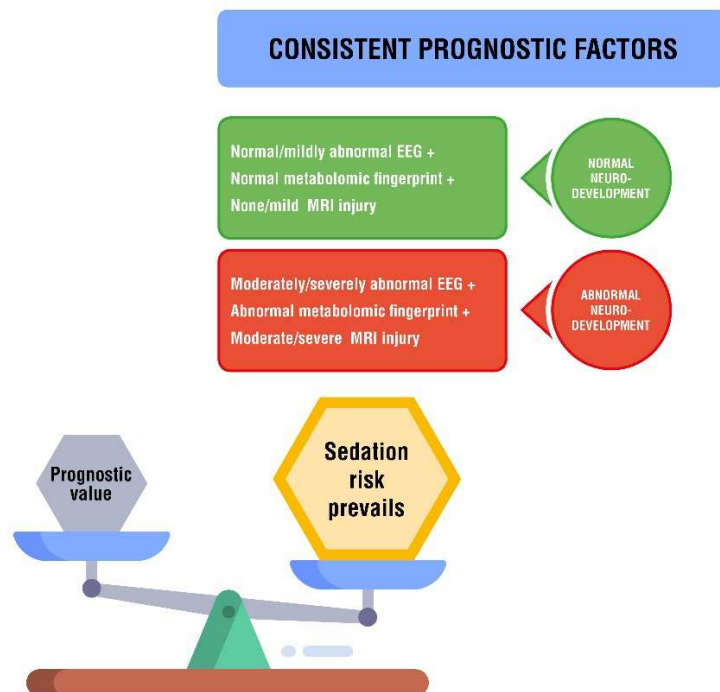

# LATE MRI INDICATION

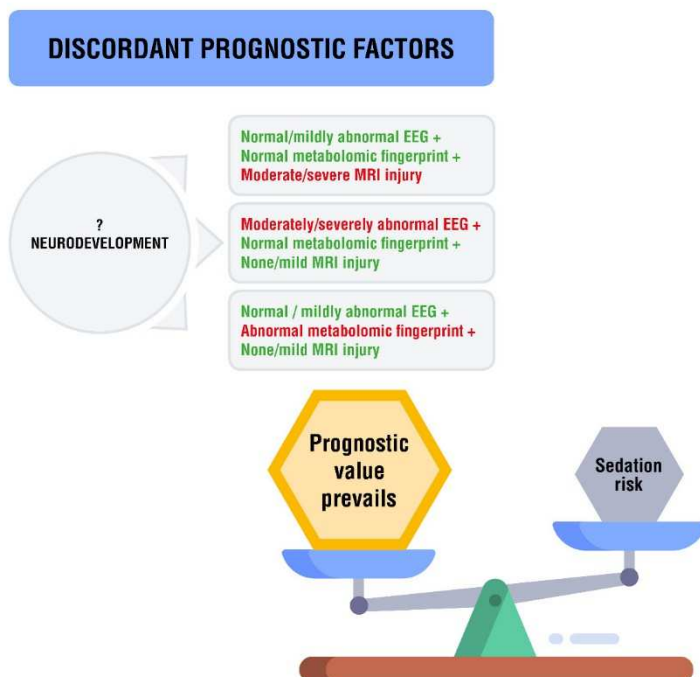

Supplement: Supplementary file 1 — Supplementary Material 1: Figure 1: chart for the indication of late MRI in HIE. This hypothetical conceptual chart aims to assist clinicians in assessing the need for a follow-up late brain MRIby integrating findings fromEEG background analysis, neonatal metabolomic profile, and early brain MRI [file 12967_2025_6714_MOESM1_ESM.pdf]
