# Supplementary material for: Urinary metabolome at birth in patients with hypoxic–ischemic encephalopathy treated with therapeutic hypothermia and long-term neurodevelopmental outcomes: a 7-year follow up
Source: J Transl Med. 2025 Nov 24;23:1345. doi: 10.1186/s12967-025-06714-w (PMC12645678; doi:10.1186/s12967-025-06714-w)
Supplement: Supplementary file 2 — Supplementary Material 2 [file 12967_2025_6714_MOESM2_ESM.docx]

**Supplementary Information**

**Statistical data analysis**

Data representing the characteristics of the recruited newborns were analysed by t-test or Mann-Whitney test for continuous normally or non-normally distributed data, respectively, and by Fisher’s exact test for categorical variables. A significance level α=0.05 was assumed. Normality of the data was assessed by Shapiro-Wilk test (p>0.10).

A one-to-one matching procedure was applied to extract sub-groups of newborns without significant differences in the demographic and clinical perinatal data. The procedure was an iterative procedure where at each step a pair of subjects, one belonging to the favourable outcome group and the other to the adverse outcome group, was selected on the basis of the minimum Euclidean distance calculated using the scaled perinatal variables. The procedure stopped when a significant difference in the demographic perinatal variables was observed. We assumed a significant level α=0.05.

Multivariate data analysis was applied to investigate the metabolomics data. Specifically, Principal Component Analysis (PCA) was used for outlier detection applying the T2 and the Q-test, whereas orthogonally constrained PLS for classification (oCPLS2C) with stability selection [1,2,3] for discovering the set of the most relevant features able to distinguish the groups of patients under investigation.

PLS models were constrained in order to generate score structures orthogonal to gestational age and birth weight because urinary metabolome is very sensitive to these two characteristics, and data projection may be driven along unsuitable directions if suitable constraints are not applied.

In stability selection, 200 sub-sets were randomly extracted sampling the observations and the features by Binary Matrix Sampling with probability equal to 0.8, while feature selection was based on Variable Influence on Projection (VIP), choosing at each run the optimal number of score components of the oCPLS2C model on the basis of the first maximum of the Matthew’s correlation coefficient (MCC) calculated by 5-fold cross-validation. At each run of sub-set selection, the oCPLS2C model obtained by feature selection was used to predict the unselected observations in order to calculate the out-of-bag MCC. A significance level α=0.05 was assumed to select the most relevant features that were included in the optimal subset of features.

Thus, the optimal subset was used to build the oCPLS2C model considered to study the relationships between group of patients and urinary metabolome. Repeated 5-fold cross-validation with 20 repetitions and permutation test on the group (500 random permutations) were applied to assess model reliability and to estimate the optimal number of score components of the model. Specifically, the number of components that generates the model showing the first maximum of MCC in cross-validation (MCCcv) under the condition to pass the permutation test assuming α=0.05, was considered as the optimal number of components to use.

Moreover, the optimal subset of features was investigated by Multiple Linear Regression (MLR) considering group of patients, gestational age and birth weight as factors and the metabolite concentration as response.

Data analysis was performed using in-house R-functions implemented by R 4.0.4 platform (R Foundation for Statistical Computing).

**References**

1. Stocchero M, De Nardi M, Scarpa B. PLS for classification. Chemometr Intell Lab Syst. 2021; 216:104374.

2. Stocchero M. Relevant and irrelevant predictors in PLS2. J. Chemometr. 2020; 34 e3237.

3. Stocchero M, Riccadonna S, Franceschi P. Projection to latent structures with orthogonal constraints for metabolomics data. J. Chemometr. 2018; 32 e2987.
